# The influence of spasticity on goniometric range of motion measurement in children with cerebral palsy

**DOI:** 10.1186/s12891-026-09659-3

**Published:** 2026-03-04

**Authors:** Olof Lindén, Katarina Lauruschkus, Philippe Wagner, Gunnar Hägglund, Henrik Lauge-Pedersen

**Affiliations:** 1 Department of Clinical Sciences, Lund University, Lund, Orthopedics Sweden; 2 Department of Orthopedics, Skane University Hospital, Lund, Sweden; 3 Department of Health Sciences, Faculty of Medicine, Lund University, Lund, Sweden; 4 Faculty of Health Sciences, Kristianstad University, Kristianstad, Sweden; 5 Centre for Clinical Research, Uppsala University, Region Västmanland, Västerås, Sweden

**Keywords:** Children, Cerebral palsy, Spasticity, Goniometer, Range of Motion, Reliability, Bias

## Abstract

**Introduction:**

Spasticity may introduce bias in passive range of motion (ROM) measurements in children with cerebral palsy. This study aimed to explore the impact of spasticity on the reliability and potential measurement bias of goniometric assessments of ankle dorsiflexion and knee extension in children with unilateral spastic cerebral palsy (USCP).

**Methods:**

Thirty-two children aged 6–17 years with unilateral spastic cerebral palsy were included. Two blinded investigators measured ROM on the spastic and contralateral sides using a two-axis goniometer, and spasticity was graded with the Modified Ashworth Scale. The primary outcome was the between-side difference in inter-examiner measurement bias, analysed using paired *t*-tests and confidence intervals. Secondary outcomes—agreement and reliability—were assessed using Bland–Altman plots and intraclass correlation coefficients (ICC).

**Results:**

No clinically meaningful differences in ROM measurement bias were found between the spastic and contralateral sides for ankle dorsiflexion (extended knee: mean difference − 0.22°, 95% CI: − 2.53 to 2.09; flexed knee: 0.78°, 95% CI: − 3.57 to 2.01), knee extension (0.31°, 95% CI: − 1.63 to 1.01), or hamstrings angle (0.50°, 95% CI: − 2.53 to 3.53). ICC indicated high inter-rater reliability.

**Conclusions:**

These findings support the use of goniometry as a reliable tool for assessing joint mobility in clinical and research settings. Results were reliable for measurements on a spastic and contralateral limb and no clinically significant bias was found.

**Supplementary Information:**

The online version contains supplementary material available at 10.1186/s12891-026-09659-3.

## Introduction

The risk of joint contractures in the lower limbs is a significant concern in growing children with cerebral palsy (CP). Assessing joint mobility is essential for physicians and physiotherapists when evaluating and treating these children. Early identification of a decreasing passive range of motion (ROM) is crucial for enabling successful treatment and optimizing long-term outcomes [1]. The development of contractures can sometimes progress slowly, requiring a measurement method that is both accurate and reliable.

The primary method for measuring joint ROM is the use of a manual goniometer, although techniques using smartphones or other electronic devices also have been demonstrated [2–4]. Previous studies indicate that traditional goniometry is generally reliable for measuring both ankle and knee ROM in individuals with and without spastic muscles [2–7], even if McDowell et al. reported considerable variability of goniometric measurements in ambulatory children with spastic cerebral palsy [8]. Most previous studies have examined the overall reliability across raters or groups of children with CP. However, a high reliability does not exclude a presence of systematic error or bias. During goniometric ROM assessment, small differences between examiners in applied force, or the child’s ability to relax may influence the measurement result and lead to lower or higher measured ROM in a spastic limb. The aim of this study was to investigate the impact of spasticity on potential bias. To our knowledge, this within-subject approach (spastic vs. contralateral limb) has not previously been used to specifically evaluate inter-examiner agreement and potential side-related measurement bias in children with unilateral spastic cerebral palsy USCP. This was achieved by using each child as their own control, comparing measurements from the spastic limb to those of the contralateral limb.

## Methods

In Sweden, almost all children with CP are connected to a local habilitation unit where regular assessments are conducted by the child’s physiotherapist through the Swedish Follow up Program for People with Cerebral Palsy (CPUP) [9]. Families of children aged 2–17 years with USCP, and living in the Skåne Region, were invited to participate in the study. The study information was given both to each child and their parents, including an easy-to-read child-appropriate version. After two weeks, a reminder was sent to all families who had not answered. Children with diagnoses other than USCP were excluded, as were those who had undergone lower extremity surgery within the past year or received botulinum toxin injections to the lower extremity within the last three months. The children who consented to participate and completed the examination were between 6 and 18 years of age at the time of assessment. All participants had USCP, diagnosed by a pediatric neurologist. USCP was defined clinically as predominant unilateral involvement; therefore, mild spasticity may also be observed in the contralateral limb without implying bilateral spastic CP [10].

**Fig. 1:**
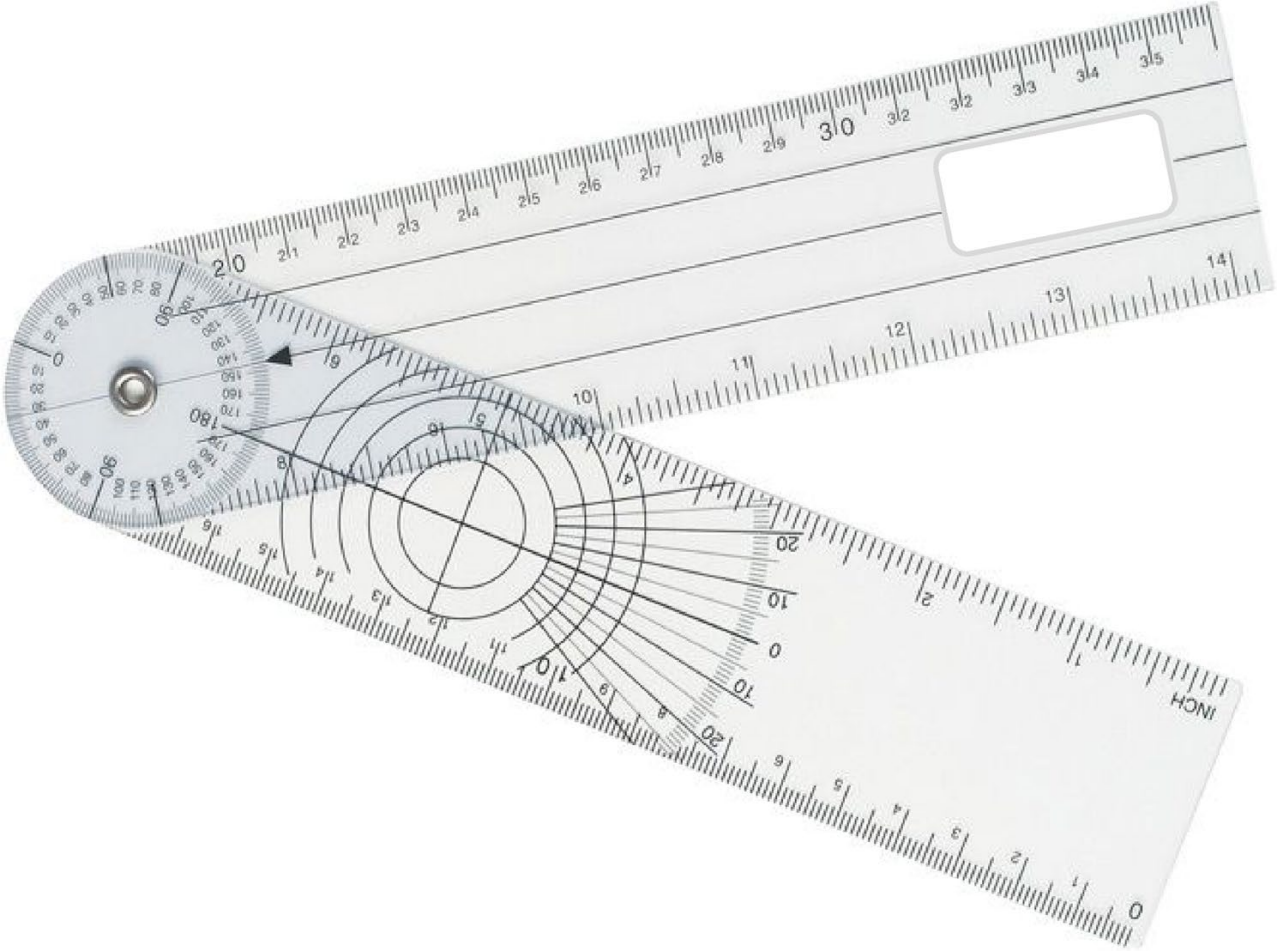
Standard goniometer

**Fig. 2:**
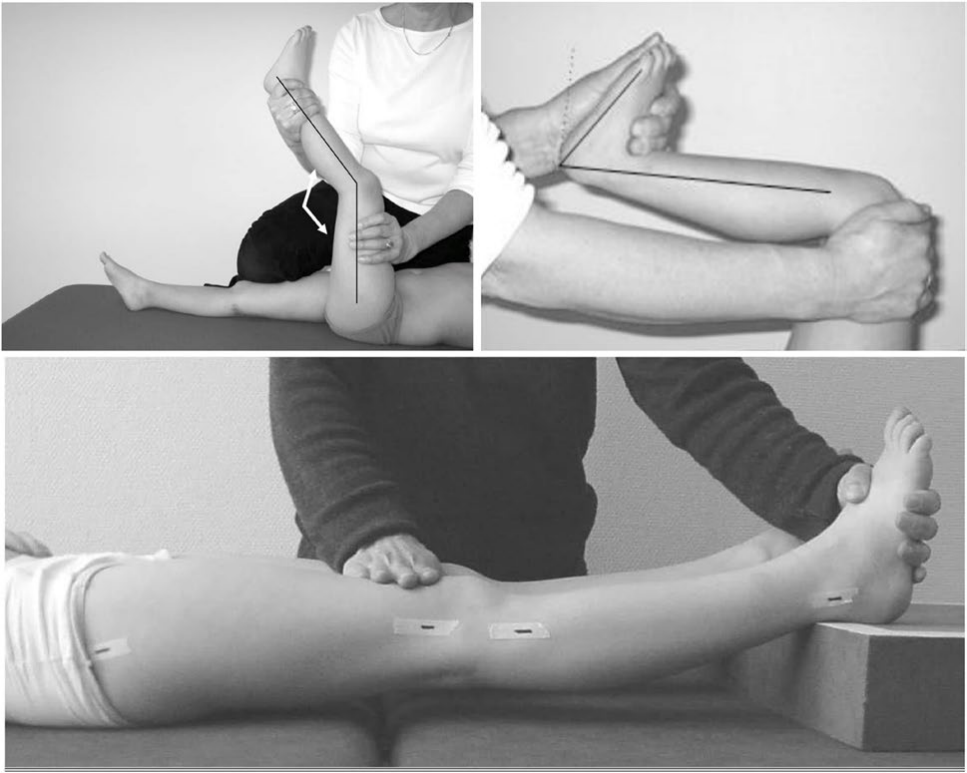
Standardized techniques were followed for measuring ROM, with the child in a supine position. The hamstrings angle was measured with the knee flexed, and ankle dorsiflexion was evaluated with the subtalar joint stabilized with flexed and extended knee. Reproduced with permission from the CPUP program. (https://cpup.se/wp-content/uploads/2023/01/FT-manual-2023.230116.pdf)

The participants were assessed in a single session lasting approximately 30 min. The order of limb assessment was not randomized or pre-specified; examiners performed the measurements according to their usual clinical routine. Although examiners were blinded to each other’s results, the clinically spastic side was typically apparent in children with USCP and effective blinding to side was therefore not feasible. ROM measurements of knee extension and ankle dorsiflexion were performed on both legs of each child by two investigators using a standard two-axis goniometer commonly used in clinical practice. Each examination was performed by a single assessor, who manually stabilized the limb and foot and then aligned the goniometer with the anatomical landmarks according to the CPUP protocol. The goniometer consists of two 20 cm arms joined by a pivot, with a graduated scale (Fig. 1). Standardized patient positioning and goniometer arm placement for measuring ROM in different joints were based on the guidelines from the CPUP manual (https://cpup.se/wp-content/uploads/2023/01/FT-manual-2023.230116.pdf) (Fig. 2).

Hamstrings angle was measured with the child positioned supine, with the hip of the measured leg flexed to 90°, while the contralateral leg was fixed in extended position. The pivot of the goniometer was placed laterally over the knee joint, with the fixed arm aligned along the femur and pointing toward the greater trochanter, and the moving arm held parallel to the anterior margin of the tibia, pointing toward the lateral malleolus.

Knee extension was measured with the child in supine position and both knees extended. The pivot of the goniometer was placed laterally over the center of rotation of the knee joint. The fixed arm was aligned with the femur, pointing toward the greater trochanter, while the moving arm was held parallel to the anterior margin of the tibia, pointing toward the lateral malleolus. Any deficit in knee extension was recorded as a negative value.

Ankle dorsiflexion was measured with the knee flexed and extended respectively, and the child in a supine position. The subtalar joint was stabilized by the examiner while intertarsal movement was inhibited by slightly inverting the forefoot. The fixed arm of the goniometer was aligned parallel to the anterior margin of the tibia, and the moving arm was positioned parallel to the lateral margin of the foot. A 90° ankle position was recorded as 0°, and dorsiflexion less than 0° was noted as a negative value.

The two assessors were a senior pediatric physiotherapist and a pediatric orthopaedic surgeon, both with more than 10 years of experience in clinical management of children with CP and in performing goniometric ROM measurements within CPUP. Data collected included age, sex, spastic side (right/left), and spasticity level according to the Modified Ashworth Scale (MAS) (Bohannon et al. 1987). The level of spasticity in plantar flexors, knee extensors and knee flexors on each side was determined by consensus between the two investigators. The investigators performed the ROM-measurements independently and were blinded to each other’s results. Each examiner performed a single ROM measurement for each joint position on each limb.

### Statistics

The primary hypotheses were based on comparing the inter-examiner measurement bias between the spastic and contralateral side across joints. For each participant, inter-examiner differences were calculated separately for each side, and the within-participant contrast between sides was tested against zero using a two-sided paired *t*-test.

Assuming a clinically relevant difference of 5 degrees, a two-sided significance level of 0.05, a power of 0.8, and a conservatively assumed standard deviation of 10 degrees (corresponding to zero correlation between sides), the required sample size was 32 participants.

To compare potential measurement bias between the spastic and contralateral sides, the differences between the two examiners’ measurements were calculated for the spastic and the contralateral side, respectively. Subsequently, the difference between these differences (i.e., the discrepancy in examiner agreement between the spastic and contralateral sides) was computed for each participant. Paired *t*-tests were then performed, but inference mainly relied on 95% confidence intervals for the mean differences; intervals entirely below the minimally clinically important difference of 5° indicated no systematic bias.

Bland–Altman plots were generated separately for the spastic and contralateral sides to assess systematic bias, proportional bias, and limits of agreement between examiners.

In addition, inter-rater reliability between the two examiners was assessed using the intraclass correlation coefficient (ICC) together with 95% confidence intervals, calculated separately for the spastic and contralateral sides. The examiners’ measurements were treated as fixed effects and the participants as random effects. ICC values were interpreted according to Koo et al., with values below 0.5 indicating poor reliability, between 0.5 and 0.75 indicating moderate reliability, and above 0.75 indicating good to excellent reliability [11].

All statistical analyses were performed using SPSS Statistics, version 28.0.

## Results

A total of 32 children with USCP participated in the study, including 17 boys and 15 girls. The mean age at assessment was 11.9 years (SD 3.9, range 6.0–18.3, one participant had turned 18 years by the time of the assessment visit), with boys averaging 11.9 years (SD 3.4, range 6.7–17.2) and girls 12.0 years (SD 4.6, range 6.0–18.3). The spastic side was the right in 22 children and the left in 10 children.

Mild spasticity in the contralateral limb was observed in approximately half of the children; however, spasticity was greater on the affected side, with the most pronounced spasticity observed in the plantar flexors (Table 1).

**Table 1:** Level of spasticity (MAS) in respective muscle investigated

Muscle and side	0	1	1+	2	3	4	Total (*n*)
Gastrocsoleus spastic side	0	6	15	8	2	1	32
Gastrocsoleus contralateral side	18	13	1	0	0	0	32
Hamstrings spastic side	6	19	7	0	0	0	32
Hamstrings contralateral side	29	3	0	0	0	0	32
Rectus spastic side	28	4	0	0	0	0	32
Rectus contralateral side	32	0	0	0	0	0	32

*MAS *Modified Ashworth scale

Ankle dorsiflexion was approximately 10° lower on the spastic side compared to the contralateral side, both with the knee extended and flexed. Knee extension and hamstrings angles were about 5° lower on the spastic side. (Table 2)

**Table 2:** Mean ROM (°) for knee and ankle measurements

Measurement	Side	Examiner 1 (mean (SD), range)	Examiner 2 (mean (SD), range)
Ankle Dorsiflexion extended knee	Spastic	6.8 (7.4), -8-23	5.2 (7.9), -10-20
Ankle Dorsiflexion extended knee	Contralateral	16.3 (6.9), 4–33	14.5 (7.6), 2–30
Ankle Dorsiflexion flexed knee	Spastic	12.0 (8.9), -4-36	12.7 (8.8), -4-35
Ankle Dorsiflexion flexed knee	Contralateral	24.0 (7.9), 10–42	24.0 (7.8), 10–40
Knee Extension	Spastic	1.2 (6.8), -10-24	1.5 (6.1), -10-22
Knee Extension	Contralateral	4.7 (6.7), -5-24	4.7 (6.2), -2-21
Hamstrings angle	Spastic	143.2 (9.0), 125–165	147.4 (9.2), 122–170
Hamstrings angle	Contralateral	146.6 (9.9), 119–168	151.2 (11.1), 118–175

*ROM *Range of motion

To explore potential side-related bias in inter-examiner agreement, the differences between the two examiners’ measurements were calculated separately for the spastic and contralateral sides. Subsequently, the difference between these differences was analyzed. Across all joint measures, no systematic side-related bias was observed, as the mean differences were close to zero and the confidence intervals included zero (Table 3). P-values are reported for completeness, but the interpretation primarily relies on the confidence intervals. Sensitivity analyses (MAS = 0 contralaterally) yielded similar point estimates; 95% CIs were within ± 5° for all outcomes except ankle dorsiflexion with the knee flexed (Supplementary Table 1).

**Table 3:** Mean difference in examiner agreement between spastic and contralateral limbs

ROM measurement	Mean Difference (degrees)	SD	95% Confidence Interval	*p*-value (Two-sided)
Ankle dorsiflexion extended knee	-0.22	6.40	-2.53, 2.09	0.848
Ankle dorsiflexion flexed knee	-0.78	7.74	-3.57, 2.01	0.572
Knee extension	-0.31	3.66	-1.63, 1.01	0.632
Hamstrings angle	0.50	8.41	-2.53, 3.53	0.739

**Fig. 3:**
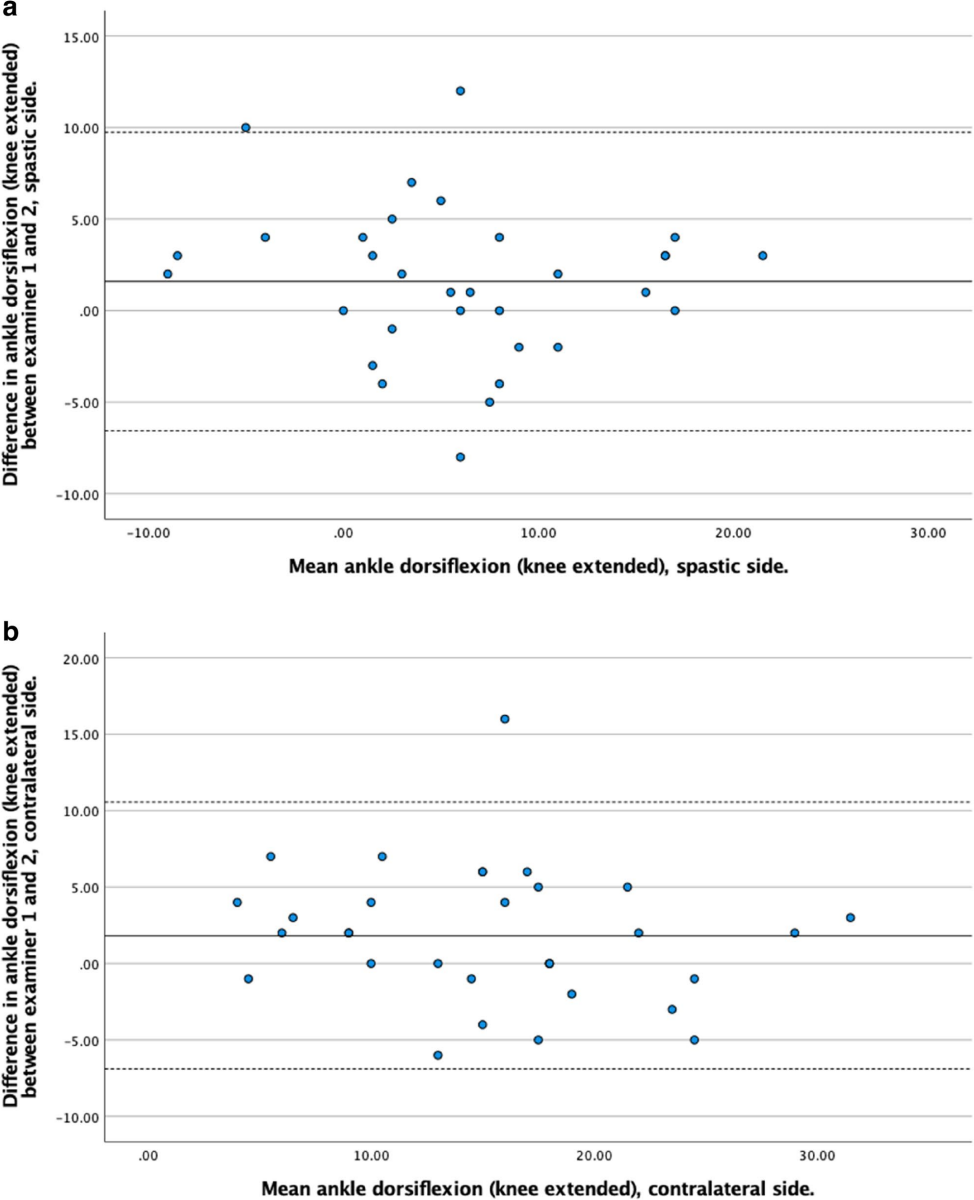
**a.** Bland-Altman plot showing the difference in ROM measurements between examiner 1 and examiner 2 (y-axis) against the mean ROM measurements (x-axis) for ankle dorsiflexion with the knee extended on the spastic side. **b.** Bland-Altman plot showing the difference in ROM measurements between examiner 1 and examiner 2 (y-axis) against the mean ROM measurements (x-axis) for ankle dorsiflexion with the knee extended on the contralateral side

**Fig. 4:**
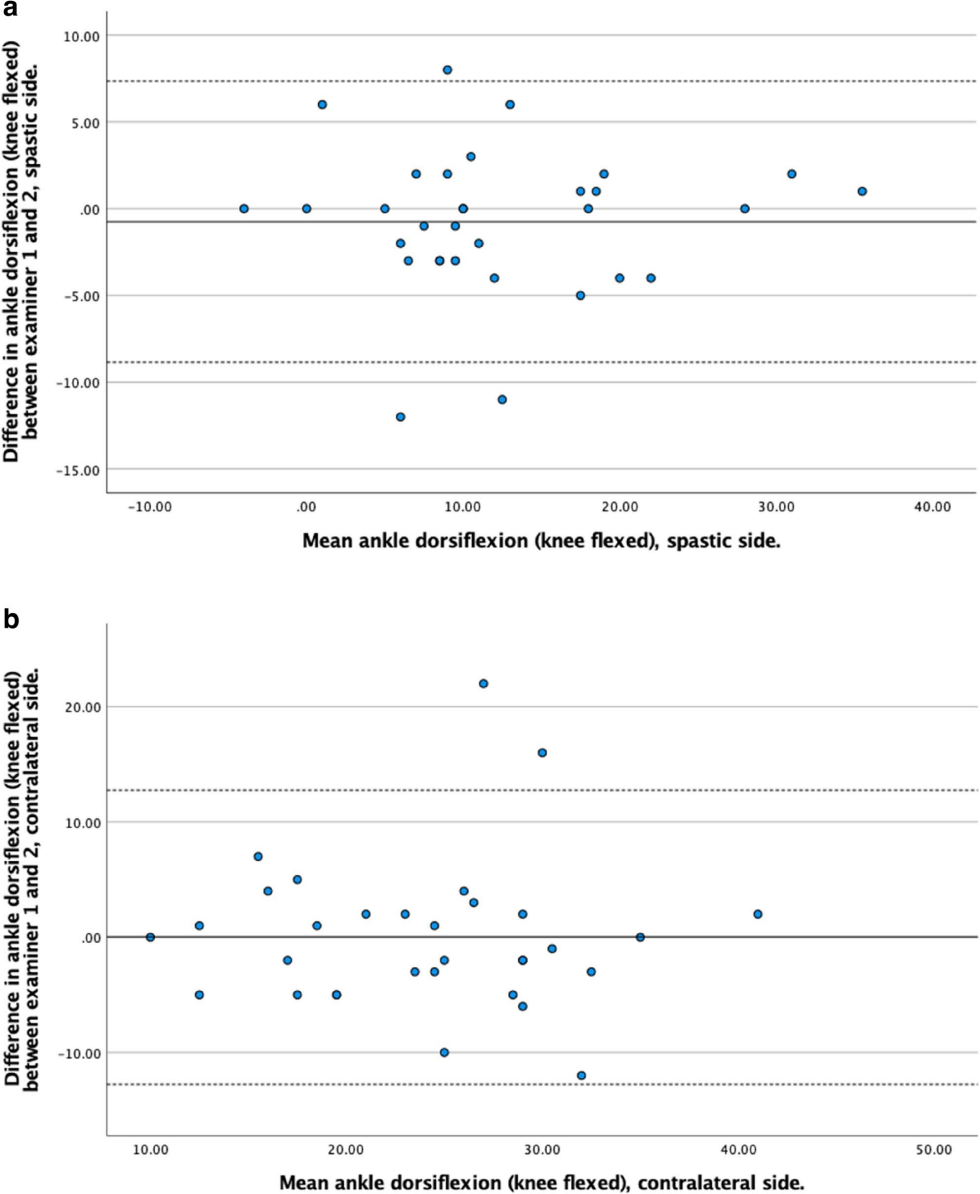
**a.** Bland-Altman plot showing the difference in ROM measurements between examiner 1 and examiner 2 (y-axis) against the mean ROM measurements (x-axis) for ankle dorsiflexion with the knee flexed on the spastic side. **b.** Bland-Altman plot showing the difference in ROM measurements between examiner 1 and examiner 2 (y-axis) against the mean ROM measurements (x-axis) for ankle dorsiflexion with the knee flexed on the contralateral side

**Fig. 5:**
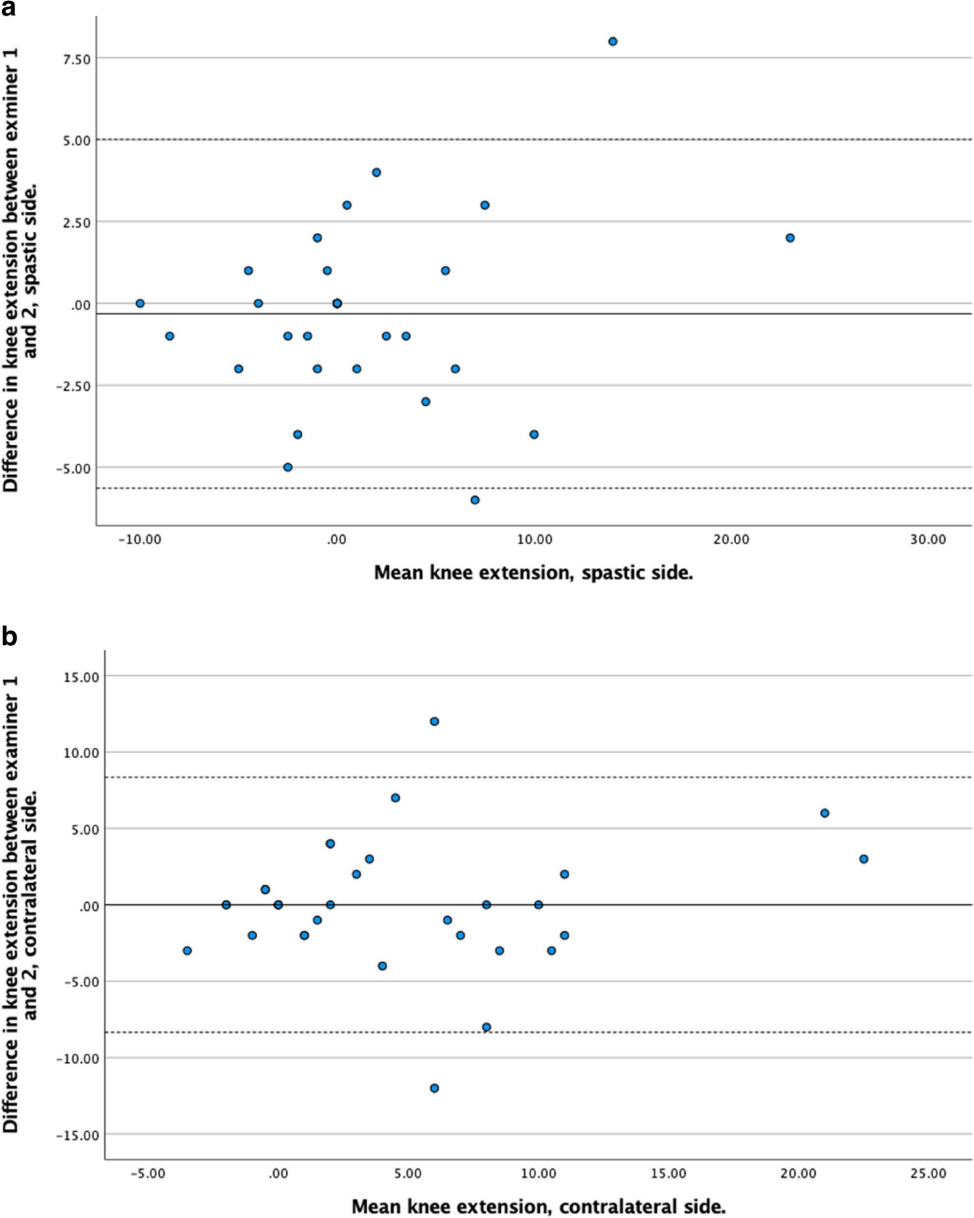
**a.** Bland-Altman plot showing the difference in ROM measurements between examiner 1 and examiner 2 (y-axis) against the mean ROM measurements (x-axis) for knee extension on the spastic side. **b.** Bland-Altman plot showing the difference in ROM measurements between examiner 1 and examiner 2 (y-axis) against the mean ROM measurements (x-axis) for knee extension on the contralateral side

**Fig. 6:**
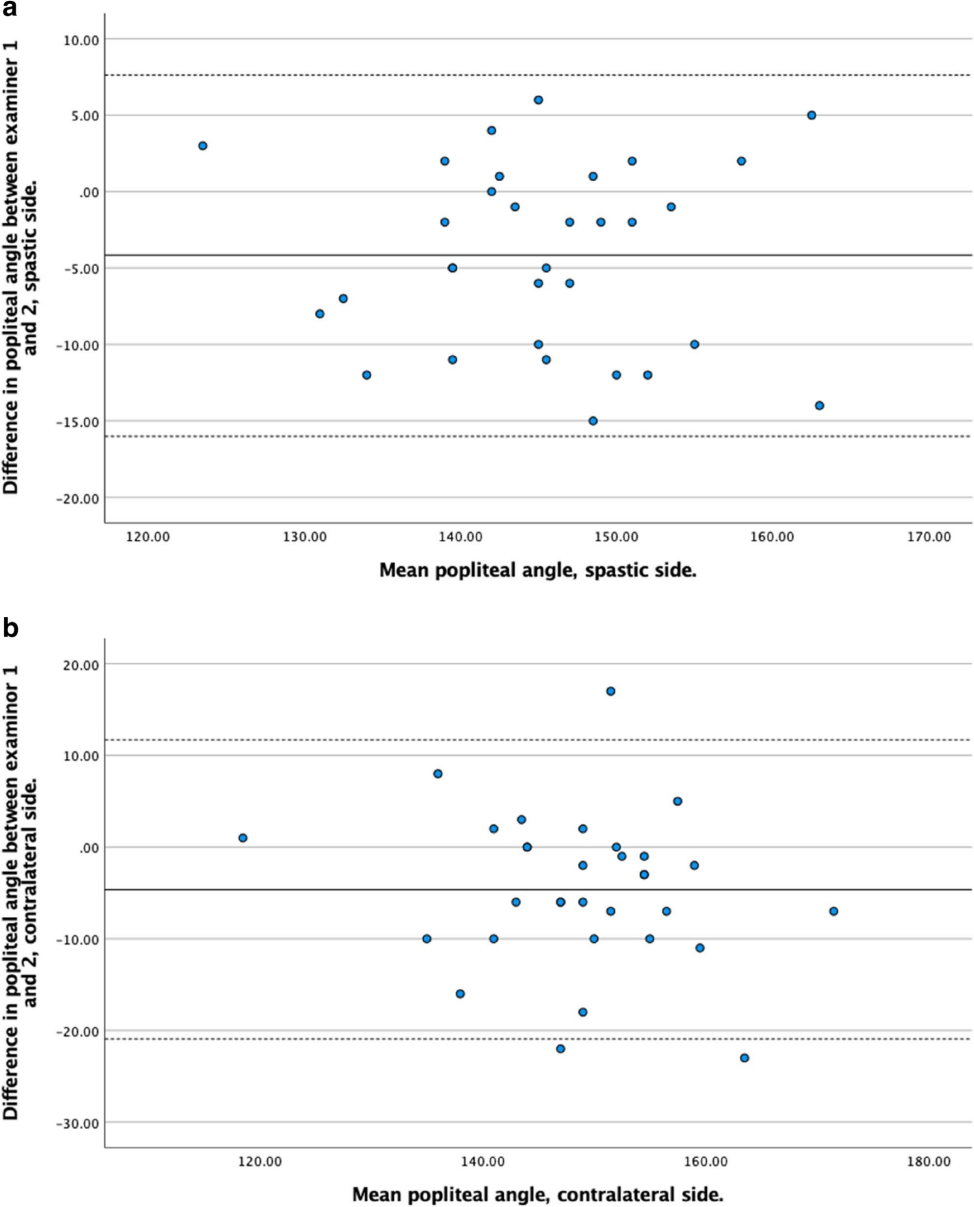
**a.** Bland-Altman plot showing the difference in ROM measurements between examiner 1 and examiner 2 (y-axis) against the mean ROM measurements (x-axis) for hamstrings angle on the spastic side. **b.** Bland-Altman plot showing the difference in ROM measurements between examiner 1 and examiner 2 (y-axis) against the mean ROM measurements (x-axis) for hamstrings angle on the contralateral side

The agreement between Examiner 1 and Examiner 2 was evaluated using Bland-Altman plots. Across all measurements, the mean differences between examiners were generally small, indicating good agreement. The limits of agreement varied depending on the joint, limb side, and whether the knee was flexed or extended, with wider variation observed in some measures, particularly for hamstrings angle and ankle dorsiflexion on the contralateral side. Detailed results for each measurement are presented in Figs. 3, 4, 5 and 6.

Inter-rater reliability assessed using single measures ICC, was generally good to excellent across the range of joint measurements. Agreement between examiners was strongest for knee extension and ankle dorsiflexion, particularly on the spastic side. Slightly lower levels of agreement were observed for measurements on the contralateral side, especially for ankle dorsiflexion with flexed knee and hamstrings angle. The width of some confidence intervals, however, indicated limited statistical precision for these measures (Table 4).

**Table 4:** Intraclass Correlation Coefficients (ICC) for knee and ankle ROM measurements

Ankle dorsiflexion, extended knee (spastic side)	ICC	95% CI
	0.852	0.719–0.925
Ankle dorsiflexion, extended knee (contralateral side)	0.814	0.653–0.905
Ankle dorsiflexion, flexed knee (spastic side)	0.891	0.789–0.945
Ankle dorsiflexion, flexed knee (contralateral side)	0.653	0.399–0.814
Knee extension (spastic side)	0.911	0.827–0.956
Knee extension (contralateral side)	0.782	0.599–0.887
Hamstrings angle (spastic side)	0.782	0.599–0.887
Hamstrings angle (contralateral side)	0.688	0.451–0.835

## Discussion

The main finding in this study was that no systematic side-related bias was observed in the agreement between the examiners’ ROM measurements on the spastic and contralateral sides. This result suggests that the inter-examiner measurement bias for both the spastic and contralateral sides is comparable, despite potential differences in muscle tone between the two sides. The lack of systematic discrepancy of examiner measurement bias between the spastic and contralateral sides was consistent across all ROM measures. A possible explanation for this finding is that the use of a standardized measurement protocol, together with the examiners’ clinical experience in assessing children with spasticity, facilitated consistent positioning and identification of end-range joint motion on both limbs. In addition, the stiffer, and often partially contracted, muscle on the spastic side may have provided a clearer end-point, potentially making the measurement even easier on that side. A distinctive feature of this study is the within-subject design, where each child’s contralateral limb served as a control. This approach reduces the risk of selection bias that may occur in studies comparing children with cerebral palsy to typically developing peers [7], and allows for a more robust assessment of examiner agreement.

The Bland–Altman analysis showed generally good agreement between the two examiners, with mean differences (bias) close to zero for most ROM measurements. However, the limits of agreement were somewhat wider on the contralateral side across several measures, possibly indicating lower measurement consistency compared to the spastic side, although the difference may also stem from random noise given the low sample size. This was most pronounced for ankle dorsiflexion with the knee flexed and for the hamstrings angle, suggesting that end range was more difficult to standardize in the contralateral limb. The relatively wide limits of agreement indicate that, when different clinicians repeatedly assess the same child over time, small changes in ROM are difficult to distinguish from random inter-examiner variability. Therefore, when ROM is used for individual clinical decision-making, small changes should be interpreted with caution.

Although the study was not specifically designed or powered to assess inter-rater reliability, the results in this cohort demonstrated good-to-excellent reliability for most joints, with slightly higher reliability observed for measurements on the spastic side compared to the contralateral side. The ICC values observed are comparable to those reported in similar studies evaluating joint angle reliability in children with cerebral palsy [7, 11, 12]. Slightly lower reliability was observed for some measurements, such as ankle dorsiflexion with the knee flexed on the contralateral side. This could reflect greater variability in the contralateral limb due to less pronounced stiffness compared to the spastic side. Overall, these ICC findings support reliability of using goniometric measurements for assessing joint mobility, even in children with spasticity and CP. However, given that the study was not specifically powered for ICC analysis, these results should be interpreted with caution.

The strength of this study is the inclusion of both the spastic and contralateral sides within the same individuals, which eliminates inter-individual variability and potential confounders such as age, overall motor function, and growth-related changes. This approach provides a more controlled comparison of joint angle differences and measurement reliability, making the results more robust than studies comparing children with cerebral palsy to typically developing peers.

However, certain limitations must be acknowledged. Mild contralateral increased tone was common (Table 1), which does not necessarily indicate misclassification because unilateral spastic CP is defined by predominant unilateral motor involvement. This could reduce the contrast between limbs; however, sensitivity analyses restricted to children with MAS = 0 on the contralateral side showed unchanged results, supporting the robustness of the main findings. In addition, only a single measurement per limb was obtained from each examiner. While this reflects routine clinical practice, it limits the ability to quantify within-examiner variability and may reduce the precision of agreement estimates. The protocol did not pre-specify or record the interval between repeated passive assessments by the two examiners. Because muscle tone and cooperation may fluctuate with repeated stretching, an order/time effect cannot be fully excluded. The age span is wide and may reflect differences in cooperation and neuromuscular characteristics. We did not have sufficient power to evaluate age as a modifier of inter-examiner variability and subsequent examiner agreement. Intra-examiner reliability was not formally assessed in this study, since the primary focus was on inter-examiner measurement bias and its potential side-related difference.

To the best of our knowledge, no previous studies have compared ROM measurements between the spastic and contralateral sides within individuals with USCP for the purpose of evaluating inter-examiner agreement. In the present study, no evidence of clinically relevant side-related bias was found, and the overall high inter-rater reliability across all measurements supports the use of goniometry as a robust and reliable method for assessing joint range of motion in this population.

## Supplementary Information

Supplementary Material 1.

## Data Availability

The datasets analysed during the current study are available from the corresponding author on reasonable request.
