# Supplementary material for: The influence of spasticity on goniometric range of motion measurement in children with cerebral palsy
Source: BMC Musculoskelet Disord. 2026 Mar 4;27:289. doi: 10.1186/s12891-026-09659-3 (PMC13064034; doi:10.1186/s12891-026-09659-3)
Supplement: Supplementary file 1 — Supplementary Material 1. [file 12891_2026_9659_MOESM1_ESM.docx]

**Supplementary data**

**Supplementary Table 1. Sensitivity analysis restricted to children with MAS = 0 on the contralateral (less spastic) side.**

| **ROM measurement** | **Mean Difference (degrees)** | **SD** | **95% Confidence Interval** | **p-value (Two-sided)** |
| --- | --- | --- | --- | --- |
| **Ankle dorsiflexion extended knee** | -0.50 | 6.09 | -3.53, 2.53 | 0.732 |
| **Ankle dorsiflexion flexed knee** | -2.22 | 8.91 | -6.66, 2.21 | 0.305 |
| **Knee extension** | -0.28 | 3.69 | -1.68, 1.13 | 0.691 |
| **Hamstrings angle** | 0.10 | 7.54 | -2.76, 2.97 | 0.942 |
